# A computational approach to chemical etiologies of diabetes

**DOI:** 10.1038/srep02712

**Published:** 2013-09-19

**Authors:** Karine Audouze, Søren Brunak, Philippe Grandjean

**Affiliations:** 1 Center for Biological Sequence Analysis, Department of Systems Biology, Technical University of Denmark, DK-2800 Lyngby, Denmark; 2 NNF Center for Protein Research, Health Sciences Faculty, University of Copenhagen, Denmark; 3 Institute of Public Health, University of Southern Denmark, Odense, Denmark; 4 Department of Environmental Health, Harvard School of Public Health, Boston, Massachusetts, USA

## Abstract

Computational meta-analysis can link environmental chemicals to genes and proteins involved in human diseases, thereby elucidating possible etiologies and pathogeneses of non-communicable diseases. We used an integrated computational systems biology approach to examine possible pathogenetic linkages in type 2 diabetes (T2D) through genome-wide associations, disease similarities, and published empirical evidence. Ten environmental chemicals were found to be potentially linked to T2D, the highest scores were observed for arsenic, 2,3,7,8-tetrachlorodibenzo-*p*-dioxin, hexachlorobenzene, and perfluorooctanoic acid. For these substances we integrated disease and pathway annotations on top of protein interactions to reveal possible pathogenetic pathways that deserve empirical testing. The approach is general and can address other public health concerns in addition to identifying diabetogenic chemicals, and offers thus promising guidance for future research in regard to the etiology and pathogenesis of complex diseases.

More than 35 million deaths per year – 60% of all global deaths – are attributed to non-communicable diseases (NCDs), including diabetes, cardiovascular disease, metabolic syndrome and chronic lung disorders^1^. In 2008, more than 180 million people had diabetes, and this number is expected to double by 2030. While diet, overweight, and exercise are important risk factors, new evidence suggests that environmental chemicals may contribute importantly to the pathogenesis of diabetes^2^,^3^. Genetic factors play a role as well, although each of several heterogeneities identified seems to contribute only minor risk^4^. Gene-environment interaction analysis is an option that has not yet been explored due to the very large number of chemical substances that may interact with several dozen genes involved in diabetes pathogenesis.

Emerging evidence suggests that a number of environmental chemicals may play a causative role, but this has not been screened systematically. Increased diabetes risk has been shown to result from mass food poisoning^5^, occupational exposures^6^, and associations gleaned from cross-sectional population studies^7^,^8^. Experimental studies have mainly addressed lipophilic halogenated pollutants and diabetogenicity testing is not commonly conducted, although some methodological approaches appear promising^9^. Given the magnitude of the public health problem that the diabetes epidemic represents, new approaches are needed to identify chemical exposures that may deserve attention by the research community and regulatory agencies.

In silico modeling would thus seem attractive. Our recent study of the pesticide DDT^10^ demonstrated the potential of using an integrated chemical biology approach to link environmental chemicals to possible disease outcomes. While previous studies, such as ours, examined individual compounds and identified their possible effects via possible protein interactions, we now propose to link genes known to confer risk to a particular disease to environmental chemicals through protein interactions modeled by meta-analysis of multiple data sources. This method is therefore hypotheses generating and does not constitute formal testing of diabetogenicity. Confirmation of hypothetical effects require experimental testing targeted toward substances identified by the in silico approach.

The proposed methodology involves integration of three layers of information. Figure 1 shows how the different types of data are integrated: (1) a genome-wide association (GWA) layer that links single-nucleotide polymorphisms (SNP) to the disease; (2) a disease similarity layer that integrates information of diseases similar (in term of genes) to the disease of interest; and (3) a literature-based approach to identify chemicals that have shown relationship with the disease. Each of these layers involves uncertainty and incomplete data, but by integrating the total information from all three sources, we demonstrate the complementarity of the data and the usefulness in regard to identifying possible chemical causes of type 2 diabetes and the possible pathogenesis.

## Results

To evaluate the proposed meta-analysis approach in regard to a major non-communicable disease, we applied it to T2D with the aim to identify potential diabetogenic chemicals. The three different layers of evidence exploit the potential complementarities in available sources of information.

From the GWA layer, a total of 60 SNPs were extracted from the scientific literature^4^ and the Online Mendelian Inheritance in Man (OMIM) database^11^ (access as of January 2012) (Table S1). Of these genes, 54 were linked to a total of 159 chemicals in the ChemProt database^12^,^13^. Figure S1 shows a Heatmap visualization of these associations [Ploner, A. Heatplus: Heatmaps with row and/or column covariates and colored clusters. R package version 2.1.0 (2011)].

**Figure 2:**
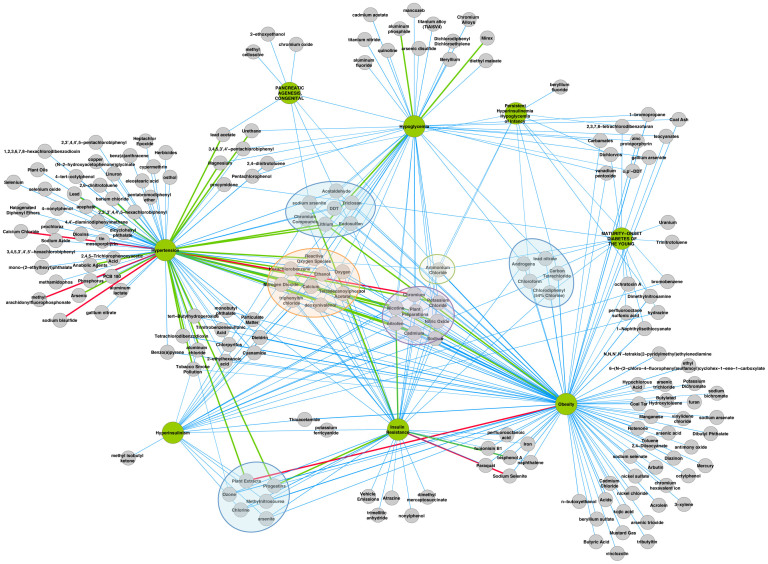
Disease layer: Disease-chemical associations. Green nodes are the eight diseases, which have common genes to T2D (from the human diseasome). Chemicals (grey nodes) are connected to at least one of these diseases (data from CTD, only score > 5). The edges between a chemical and a disease represent the evidence e.g. blue edge is literature-based, red edge is therapeutic and green edge is marker/mechanism. The six clusters show the chemicals the most connected to diseases. The green cluster contains only one chemical linked to six diseases. The purple cluster group the chemicals having associations to five diseases. The orange cluster shows association between chemicals and four diseases, and the blue ones between chemicals and three diseases. All other chemicals are connected to one or two diseases only.

In the disease similarity layer, 22 different diseases are connected to diabetes in the human diseasome^14^ (Table S2). We extracted information for the eight of them considered most relevant to the specific disease of interest, i.e., diseases known to be directly related to T2D, abnormal glucose metabolism and/or metabolic syndrome. From the Comparative Toxicogenomics Database (CTD) (access as of January 2012)^15^, 183 chemicals were identified with an interaction with at least one of the eight related diseases and with CTD score minimum of five. Figure 2 represents the connections between the eight diseases and the chemicals.

For the literature layer, all chemicals considered in the National Toxicology Program (NTP) review were extracted^8^ (Tables S3). This systematic and high-quality review represents the current epidemiologic and experimental evidence on associations between exposures to environmental chemicals and T2D.

After compilation of all chemicals retrieved from the three layers, a total of 262 unique chemicals were identified (Table S4). After exclusion of drugs and natural compounds, all environmental chemicals were ranked (Table 1). Among them, ten chemicals are present in all three layers. Most of these are commonly present in human exposures^16^. Some of these chemicals, such as bisphenol A and phthalates have a short elimination half-life that complicates exposure assessment and may therefore not be as relevant as chemicals that are more likely to accumulate in the body^17^. Another compound is also retrieved, dichlorodiphenyltrichloroethane (DDT), was already the focus of our previous study^10^. We focused on four remaining chemicals, persistent substances to which humans are commonly exposed, i.e., arsenic, hexachlorobenzene (HCB), perfluorooctanoic acid (PFOA), and 2,3,7,8-tetrachlorodibenzo-*p*-dioxin (TCDD).

For these four substances, additional exploration of the possible pathogenesis was carried out by extracting the curated chemical-gene-T2D interactions from the CTD database^15^. A total of 16 genes were found for arsenic, 8 genes for HCB, 65 genes for TCDD, and 27 for PFOA (Table S5). Following the identification of these possible links, their impact was evaluated with T2D and related disorders as diverse biological outcomes. For each chemical, the list of proteins was considered as a small biological network. Diseases and pathways were independently integrated in each biological network in order to identify significant enrichment of proteins. A source of protein-disease information, the GeneCards database (access as of August 2012) was used for the disease data integration^18^. Two sources of pathway information were used: the KEGG pathway database (access as of August 2012)^19^ and the Reactome database (access as of August 2012)^20^. This integrative step allows linking a chemical to human disorders and pathways via the proteins.

The analysis using the GeneCards database allowed linkage of all four chemicals to diabetes, TCDD, PFOA and arsenic being the most significantly associated chemicals (Table 2). When focusing on non-insulin dependent diabetes mellitus (NIDDM), similar associations were found for TCDD, PFOA and arsenic. Using the KEGG pathway database, the association between TCDD and Type 2 diabetes mellitus pathway is highly significant (corrected p-value of 5.29 × 10-7). Results obtained for PFOA, arsenic and HCB show less obvious links with the diabetes pathogenetic pathways. The diverse structural diversity of these chemicals is notable. Arsenic is a metalloid that occurs in different oxidation states, HCB and TCDD are chloride substituted aromatic compounds, and PFOA is a perfluorinated alkyl compound. This structural diversity may explain the difference in terms of the variety of proteins perturbed by the chemicals.

## Discussion

The present study explores the potential use of existing gene and protein databases to identify environmental chemicals that may be involved in the pathogenesis of important diseases. Type 2 diabetes is particularly useful for this study, as many genes are thought to be related to the development of this disease, and because diabetes also occurs in connection with other common diseases, for which genetic predisposition exists. While exposure to several environmental chemicals has been reported to increase the risk of developing diabetes^8^, the epidemiological evidence is limited, and no systematic studies in experimental toxicology have been carried out. Thus, the need for alternative approaches is obvious.

The use of chemical biology databases is advantageous, as hypothetical associations can be explored, whether or not such links have been examined before. However, only documented protein affinities should of course be evaluated, and the non-hypothesis driven assessment therefore does depend on the availability of basic chemical data. Also, the genes examined are the ones currently assumed to confer most of the increased risk of the disease, and other genes may be of importance but have not yet been documented. Still, the computational chemistry approach may be repeated with additional genes or protein affinities added from updates of the databases, without major costs, especially in comparison with the costs incurred in experimental toxicology studies. Nonetheless, the in silico findings must be considered hypotheses, as interactions can be agonistic or antagonistic, and because metabolism or other binding of the parent chemical may affect the likelihood of protein binding.

While we relied on the reports from the National Toxicology Program^8^,^21^, another listing of possible chemical causations is available from the Collaborative on Health and the Environment (http://www.healthandenvironment.org/tddb). Both sources emphasize that arsenic is strongly connected to T2D, as documented from studies of populations with increased arsenic exposures from contaminated drinking water^22^.

The substance that appears to be the most clearly connected to T2D is TCDD, a highly persistent environmental chemical that has been linked to T2D in numerous studies of populations exposed to elevated TCDD levels, e.g., from contaminants in the Agent Orange herbicide^7^.

HCB is a fungicide formerly used for seed treatment, though now banned. One study of adult Native Americans show a positive association with diabetes and HCB, but this study did not distinguish between diabetes type 1 and 2^22^. A study of US nurses showed that development of diabetes was associated with increased HCB concentrations in serum collected at baseline^22^. In support of HCB as a possible diabetogenic substance, the KEGG linkage to the T2D pathway via the IRS1 gene has been documented experimentally^23^.

Occupational exposure to perfluorinated alkylates is associated with an increased diabetes mortality^24^,^25^,^26^ though not uniformly so^27^. However, diabetes as a cause of death on death certificates is not a reliable way of obtaining information on diagnoses. In a general population study, serum-PFOA concentrations in adults were positively associated with their beta cell function (possibly as a sign of compensation for insulin resistance)^28^. Thus, PFC-induced insulin insensitivity deserves attention^8^.

Our findings show excellent agreement between three sources of information and therefore suggest a reasonable robustness of the in silico assessment of environmental chemical causations of a common non-communicable disease. The calculations are non-demanding and unbiased, although they must rely on the experimental evidence available, thus perhaps overlooking causal associations due to lack of data. However, the chemical databases are now of considerable size and are likely to provide more extensive coverage, as compared to incomplete epidemiological information. Likewise, toxicological testing for diabetogenicity is not a required component of routine chemical testing, and current knowledge on possible chemical diabetes etiologies is therefore deficient. Thus, as already recommended by a National Research Council committee^29^, computational modelling should be considered an integral part of the toxicology testing for the future. Our findings suggest that such approaches may be useful in the exploration of the pathogenesis of complex diseases, such as type 2 diabetes.

## Methods

### Data sources

For the GWA layer, we included accepted common variants [minor allele frequency (MAF) above 5%] associated with diabetes, as extracted from recent publications^4^. In addition, we included genes listed in the Online Mendelian Inheritance in Man (OMIM) database^11^. From ChemProt, a disease chemical biology database^12^,^13^, a list of environmental chemicals annotated to the selected SNPs was obtained, and the associations were illustrated by using Heatmaps [Ploner, A. Heatplus: Heatmaps with row and/or column covariates and colored clusters. R package version 2.1.0 (2011)]. The ChemProt database is a compilation of experimental data, which allows prediction of new chemical-protein interactions. The current version contains known chemical-protein annotations for more than 1,100,000 unique chemicals and more than 15,000 proteins. In the proposed study, we only used the high confidence human information, meaning only interactions when experimentally supported (binding data with IC50, gene expression levels). For the GWA layer a weight score was calculated based on the sum of genes connected to individual chemical and the total number of genes associated to T2D.

#### Disease similarity layer

To explore diseases genetically linked to T2D, we retrieved records from the human disease network^14^. Chemicals linked to the most relevant diseases associated with T2D were explored using the Comparative Toxigenomics Database (CTD)^15^. The scores for the disease similarity layer were generated in a similar way described above for the GWA layer. All chemical-disease links known as a marker or a therapeutic agent in CTD were initially kept. However, in order to reduce noise and to focus on the most relevant information, only chemical-disease data with a CTD inference score above five were considered for the chemical-disease association inferred via curated gene interaction. The inference score in CTD reflects the degree of similarity between CTD chemical–gene–disease networks and a similar scale-free random network. Many biological networks, such as disease and metabolic networks, have been shown to be scale-free random networks^30^. Thus, the score takes into account the connectivity of the chemical, disease and each of the genes used to make the chemical disease inference. The higher the score, the more likely the inference network has a non-uniform connectivity as observed in scale-free random networks. Filters (scripting) have been used to avoid unclear association, if present. For example associations such “chemical X does not affect protein Z′ and compound A co-treated with compound B affect protein Z′” were not taken into consideration.

For the literature-based layer, we used a recent authoritative literature review^8^. In this review, the authors listed environmental exposures that have been linked to T2D, as revealed by a keyword-search based strategy to identify relevant epidemiological studies. In the replication, all chemicals initially identified in the NTP review were recognized.

#### Integration of evidence layers

To identify relevant environmental pollutants, we excluded drugs and natural compounds. A combined (mean) score was calculated based on both computational scores (GWAS and disease similarity) by adding up both scores, and dividing the total by the number of scores. To integrate the literature information, we used a binary scoring scheme, i.e., 1 if the association chemical-T2D was present, and 0 if the association was absent. The chemicals were then ranked according to their combined score, and they were kept as potential candidates if documented in the epidemiological literature.

#### Systems biology

For environmental chemicals widely prevalent in human exposures^17^, we examined their curated interactions and gene/protein linkages extracted from the CTD accessed of August 2012. These data were manually processed to keep only relevant and unique information. Each protein network (one for each chemical) was used for disease and pathways enrichment (Supplementary Material and Methods and Table S6). Human disease information was extracted from the GeneCards database, a comprehensive resource for gene-related information^18^, which contains a total of 5515 genes associated to diseases. In GeneCards, 206 genes are linked to diabetes mellitus, and 228 to NIDDM. We also determined the enriched terms among pathways using the KEGG and Reactome databases^19^,^20^. Reactome contains information for 5283 genes, and among them 309 are connected to the diabetes pathways, while KEGG includes 6176 genes, of which only 48 are associated with diabetes. Gene-disease and gene-pathway relationships were independently evaluated. P-values were calculated using hypergeometric testing with Bonferroni adjustment for multiple testing. To visualize chemicals interacting with selected diseases, networks were constructed using Cytoscape^31^.

## Author Contributions

Conceived and designed the experiments: K.A. and P.G. Performed and analyzed the experiments: K.A. and P.G. Wrote the paper: K.A., S.B. and P.G.

## Supplementary Material

Supplementary Information supplementary information

## Figures and Tables

**Figure 1:**
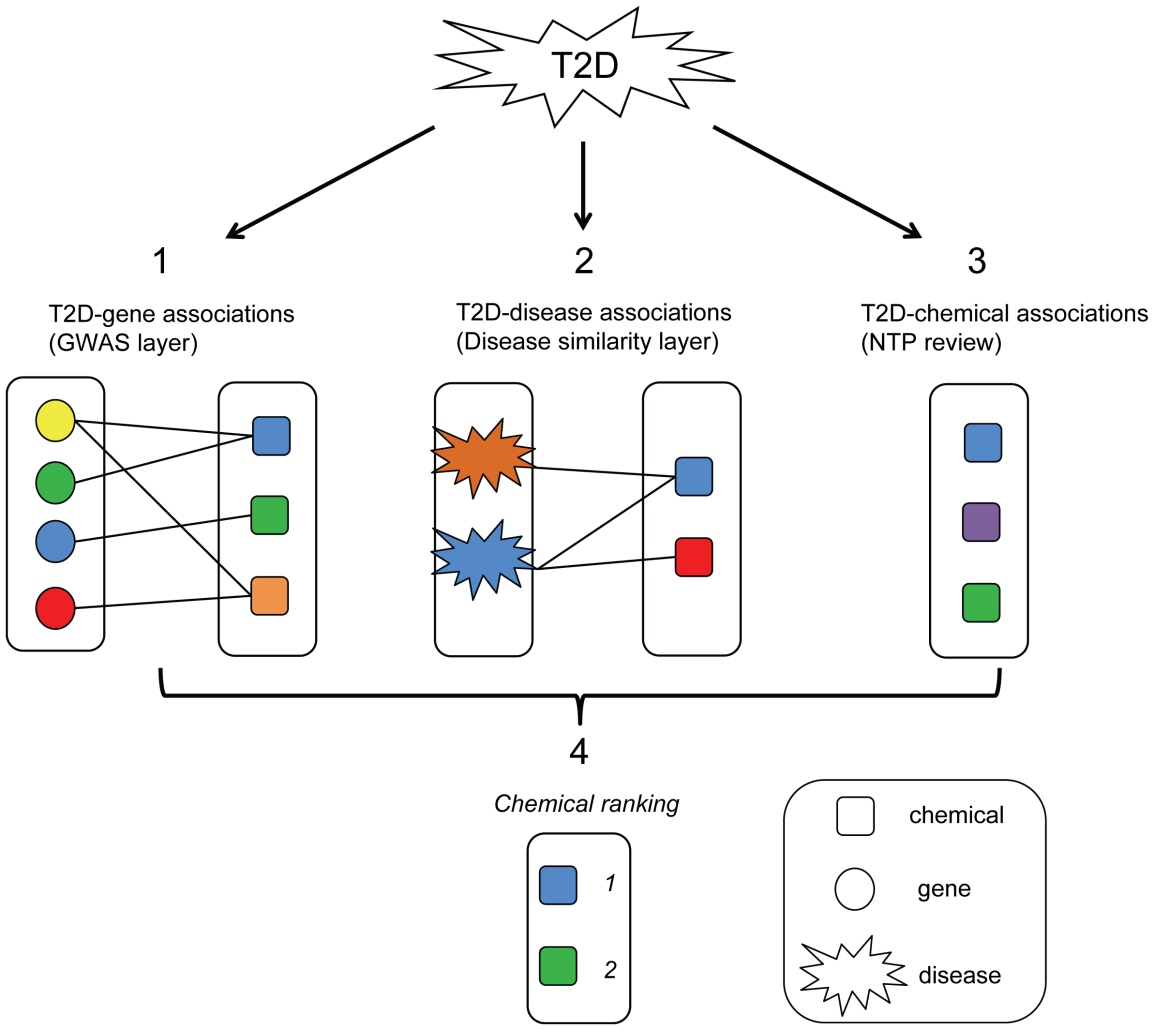
Workflow of the meta-analysis approach for identifying chemicals connected to Type II diabetes (T2D). Three data sources represent evidence layers (1–3), which allow ranking chemicals to prioritize chemicals likelihood to be involved in T2D.

**Table 1:** Ten chemicals with the strongest links to diabetes (including all three layers of information). D Score is from the disease similarity layer, GWAS score is based on SNPs information, the Combined score includes both computational layers, and the NTP evidence relies on literature documentation from a recent published review

	D score	GWAS score	Combined score	NTP evidence*
**TCDD**	0.250	0.574	0.412	1
**HCB**	0.500	0.019	0.259	1
**Bisphenol A**	0.250	0.167	0.208	1
**DDT**	0.375	0.019	0.197	1
**PFOA**	0.250	0.130	0.190	1
**PFOS**	0.250	0.130	0.190	1
**MBP**	0.250	0.019	0.134	1
**Arsenic**	0.125	0.111	0.118	1
**Dioxins**	0.125	0.019	0.072	1
**MEHP**	0.125	0.019	0.072	1

*1 if the association T2D-chemical has been reported in the literature.

**Table 2:** Disease and pathway enrichment, *p* values and genes

DISEASE		
	GeneCards (diabetes mellitus)	GeneCards (niddm)
Arsenic	5.281e-06	0.0076
	(9 genes: GCK;HMOX1;LEP;LEPR;NFKB1;PPARA;TNFRSF1A;CAT;ADIPOQ)	(7 genes: PPARGC1A;GCK;LEP;LEPR;PPARA;CAT;ADIPOQ)
PFOA	2.451e-08	2.824e-09
	(12 genes: CPT1A;GCK;HMOX1;LEPR;NFKB1;PPARA;PPARG;SLC2A2;TNFRSF1A;C3;UCP2;CAT)	(13 genes: PPARGC1A;CPT1A;GCGR;GCK;GCKR;GPD2;LEPR;LIPC;PPARA;PPARG;SLC2A2;UCP2;CAT)
TCDD	1.904e-19	2.785875e-18
	(26 genes: CPT1A;EDN1;AKT2;GCK;HMOX1;HNF4A;HP;IRS1;KCNJ11;LEP;LEPR;NFKB1;ENPP1;PPARA;PPARG;RETN;PTPN1;SLC2A1;SLC2A2;SLC2A4;TNFRSF1A;C3;UCP2;WFS1;CAT;ADIPOQ)	(26 genes: PPARGC1A;CPT1A;EDN1;GCK;GCKR;GPD2;HNF4A;IRS1;KCNJ11;LEP;LEPR;LIPC;PAX4;ENPP1;PPARA;PPARG;RETN;PTPN1;SLC2A1;SLC2A2;SLC2A4;TCF7L2;UCP2;CAT;IRS2;ADIPOQ)
HCB	0.017	n.s.
	(5 genes: HMOX1;HP;IRS1;TNFRSF1A;CAT)	(3 genes: IL6;IRS1;CAT)

PATHWAY		
	Reactome (diabetes pathway)	KEGG (Type II diabetes mellitus)
Arsenic	n.s. (1 gene: ATF3)	n.s. (2 genes: GCK;ADIPOQ)
PFAO	n.s. (1 gene: ATF3)	n.s. (2 genes: GCK;SLC2A2)
TCDD	n.s. (2 genes: ATF3;WFS1)	5.292e-07
		(8 genes: GCK;HK1;IRS1;KCNJ11;SLC2A2;SLC2A4;IRS2;ADIPOQ)
HCB	n.d	n.s. (1 gene: IRS1)

Values = p-values corrected.

n.s. = p-val no significant n.d. = no data, no gene from HCB are associated to reactome/diabetes pathway.
