# Supplementary material for: A computational approach to chemical etiologies of diabetes
Source: Sci Rep. 2013 Sep 19;3:2712. doi: 10.1038/srep02712 (PMC3965361; doi:10.1038/srep02712)
Supplement: Supplementary Information [file srep02712-s1.pdf]

A computational approach to chemical etiologies of diabetes

Karine Audouze, Søren Brunak and Philippe Grandjean

Supplementary Information

Supplementary Material and Methods
Supplementary Figure S1
Supplementary Tables S1-S6

Supplementary Material and Methods
Systems biology approach: Disease and Pathway enrichment:

For each of the four selected chemicals, arsenic, HCB, PFOA and TCDD, a list of proteins was retrieved using curated and filtered data from the CTD database. These proteins are known to be associated to the chemicals via experimental data, as, for example, the link PFOA- human PPARgamma receptor is established by the demonstration that PFOA is a partial agonist and binds to the receptor (Vanden Heuvel *et al.,* Toxicol. Sci. 2006 92(2): 476-89). In our approach each protein list was considered as a protein complex. The four chemical-related protein complexes were analyzed independently. We performed a disease enrichment using the GeneCards database and pathways enrichment using Reactome and KEGG databases.

Accordingly, for example, PFOA was connected to 27 proteins, which represent the PFOA-related protein complex. This complex was used for disease and pathway enrichment using scripts in R. Only 23 proteins were retrieved in GeneCards, of which 12 were associated with diabetes mellitus. For comparison, 206 genes on GeneCards are known to be associated to diabetes mellitus among the 5515 proteins covered (see Table S6 for details for each chemical).
To assess the information about the potential link between a chemical and a disease we calculated p-values using hypergeometric testing. The p-values are related to the number of proteins connected to a specific disease. We then used a Bonferroni correction to adjust the p-values according to the number of disease or pathway present in the databases used.

Supplementary Figure

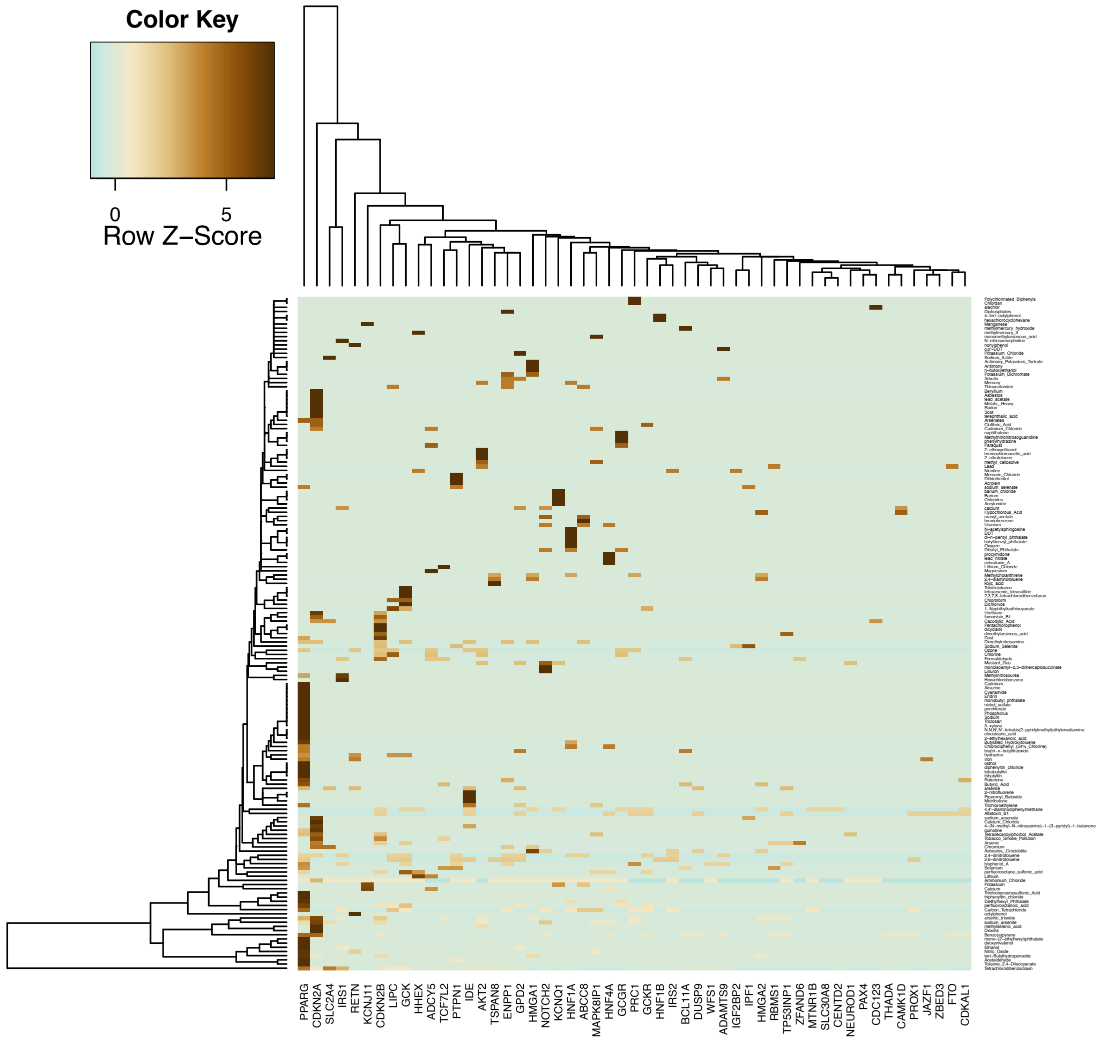

Figure S1: Heatmap representing chemicals connected to genes. Light green/blue when no chemical-gene associations are known, yellow when the associations have been found in one source of information e.g. one publication to dark brown, when associations are present in various sources of information (several publications, databases…).

Supplementary Tables

Table S1: List of the 60 SNPs extracted from literature and OMIM.

**Literature (Grarup)**	**OMIM**
ABCC8	ABCC8
ADAMTS9	-
ADCY5	-
-	AKT2
BCL11A	-
CAMK1D	-
CDC123	-
CDKAL1	CDKAL1
CDKN2A	-
CDKN2B	-
CENTD2	-
CHCHD9	-
DGKB	-
DUSP9	-
-	ENPP1
FTO	-
GCK	-
-	GCGR
-	GCK
GCKR	-
-	GPD2
HHEX	-
-	HMGA1
HMGA2	-
HNF1A	HNF1A
HNF1B	HNF1B
-	HNF4A
IDE	-
IGF2BP2	IGF2BP2
-	IPF1
IRS1	IRS1
-	IRS2
JAZF1	-
KCNJ11	KCNJ11
KCNQ1	-
KLF14	-
-	LIPC
-	MAPK8IP1
MTNR1B	-
-	NEUROD1
-	NIDDM3
-	NIDDM4
NOTCH2	-
-	PAX4
PPARG	PPARG
PRC1	-

PROX1	-
-	PTPN1
-	RETN
RBMS1	-
-	SLC2A4
SLC30A8	SLC30A8
TCF7L2	TCF7L2
THADA	-
TMEM195	-
TP53INP1	-
TSPAN8	-
WFS1	WFS1
ZBED3	-
ZFAND6	-

Table S2: List of diseases connected to diabetes in the human diseasome. In bold are the eight diseases considered most relevant to the specific disease of interest (T2D).

hypoglycemia
alzheimer disease
fancon-bickel syndrome
glioblastoma
glomerulocyste kidney disease, hypoplastic
hepatic adenoma
hyperinsulinism
hypertension
immunodysregulation, polyendocrinopathy, and enteropathy, X-linked
insulin resistance
leprechaunism
lipodystrophy
MODY
myocardial infarction
obesity
ossication of the posterior longitudinal spinal ligaments
pancreatic agenesis
persistant hyperinsulinemia of infancy
rabson-mendenhall syndrome
renal tubular dysgenesis
rheumatoid arthritis
systemic lupus erythematosus

Table S3: List of the 39 chemicals extracted from the NTP review. These chemicals are retrieved from the experimental and epidemiological literature and are known to be associated to T2D.

1,2,3,4,6,7,8-HpCDD
1,2,3,4,7,8-HxCDD
A1260
Agent Orange
arsenic
b-HCH
bisphenol A
DDD
DDE
DDT
dieldrin
dioxins
HCB
heptachlor epoxide
lidane
mirex
OCDD
organochlorines
organotins
oxychlordane
PBB153
PBBs
PBDE100
PBDE153
PBDE28
PBDE47
PBDE99
PBDEs
PCB153
PCBs dioxin like
PCBs non dioxin like
PCDD/PCDF
PFHS
PFNA
PFOA
PFOS
phthalates
TCDD
trans-nonachlor

Table S4: List of all unique chemicals identified in this study with their scores. The D Score is from the disease similarity layer, the GWAS score is based on SNPs

information, and the Combined score is the average of the two. The NTP score relies on literature documentation.

	disease score	SNP score	Combined score	NTP score
Ammonium Chloride	0.750	0.556	0.653	0
TCDD	0.250	0.574	0.412	1
Chromium	0.625	0.074	0.350	0
Nitric Oxide	0.625	0.037	0.331	0
Cadmium	0.625	0.019	0.322	0
Nicotine	0.625	0.019	0.322	0
Potassium Chloride	0.625	0.019	0.322	0
Sodium	0.625	0.019	0.322	0
nitrofen	0.625	0.000	0.313	0
Plant Preparations	0.625	0.000	0.313	0
Tetradecanoylphorbol Acetate	0.500	0.074	0.287	0
Calcium	0.500	0.056	0.278	0
Ethanol	0.500	0.056	0.278	0
Benzo(a)pyrene	0.250	0.296	0.273	0
Ozone	0.375	0.167	0.271	0
sodium arsenite	0.375	0.167	0.271	0
deoxynivalenol	0.500	0.019	0.259	0
HCB	0.500	0.019	0.259	1
Oxygen	0.500	0.019	0.259	0
triphenyltin chloride	0.500	0.019	0.259	0
Carbon Tetrachloride	0.250	0.259	0.255	0
Nitrogen Dioxide	0.500	0.000	0.250	0
Reactive Oxygen Species	0.500	0.000	0.250	0
2,4-dinitrotoluene	0.250	0.241	0.245	0
arsenite	0.375	0.093	0.234	0
Chlorine	0.375	0.093	0.234	0
Chlorodiphenyl (54% Chlorine)	0.375	0.056	0.215	0
bisphenol A	0.250	0.167	0.208	1
Chloroform	0.375	0.037	0.206	0
Lithium	0.375	0.037	0.206	0
Methylnitrosourea	0.375	0.037	0.206	0
Dimethylnitrosamine	0.250	0.148	0.199	0
Acetaldehyde	0.375	0.019	0.197	0
DDT	0.375	0.019	0.197	1
lead nitrate	0.375	0.019	0.197	0
Triclosan	0.375	0.019	0.197	0
4,4'-diaminodiphenylmethane	0.125	0.259	0.192	0
PFOS	0.250	0.130	0.190	1
PFOA	0.250	0.130	0.190	1
Androgens	0.375	0.000	0.188	0
Chromium Compounds	0.375	0.000	0.188	0
Endosulfan	0.375	0.000	0.188	0
Plant Extracts	0.375	0.000	0.188	0
Progestins	0.375	0.000	0.188	0
tert-Butylhydroperoxide	0.250	0.093	0.171	0
2,6-dinitrotoluene	0.125	0.204	0.164	0
hydrazine	0.250	0.074	0.162	0
Sodium Selenite	0.250	0.074	0.162	0
arsenic trioxide	0.125	0.185	0.155	0
Iron	0.250	0.056	0.153	0
Thioacetamide	0.250	0.056	0.153	0
Tobacco Smoke Pollution	0.250	0.056	0.153	0
1-Naphthylisothiocyanate	0.250	0.037	0.144	0
fumonisin B1	0.250	0.037	0.144	0
Paraquat	0.250	0.037	0.144	0
Urethane	0.250	0.037	0.144	0
2-ethylhexanoic acid	0.250	0.019	0.134	0

2,3,7,8-tetrachlorodibenzofuran	0.250	0.019	0.134	0
bromobenzene	0.250	0.019	0.134	0
Cyanamide	0.250	0.019	0.134	0
Dichlorvos	0.250	0.019	0.134	0
lead acetate	0.250	0.019	0.134	0
Magnesium	0.250	0.019	0.134	0
monobutyl phthalate	0.250	0.019	0.134	1
naphthalene	0.250	0.019	0.134	0
o,p'-DDT	0.250	0.019	0.134	1
ochratoxin A	0.250	0.019	0.134	0
Pentachlorophenol	0.250	0.019	0.134	0
procymidone	0.250	0.019	0.134	0
Trinitrobenzenesulfonic Acid	0.250	0.019	0.134	0
1-bromopropane	0.250	0.000	0.125	0
3,4,5,3',4'-pentachlorobiphenyl	0.250	0.000	0.125	0
aluminum chloride	0.250	0.000	0.125	0
Carbamates	0.250	0.000	0.125	0
Chlorpyrifos	0.250	0.000	0.125	0
Coal Ash	0.250	0.000	0.125	0
Dieldrin	0.250	0.000	0.125	1
gallium arsenide	0.250	0.000	0.125	0
Isocyanates	0.250	0.000	0.125	0
Particulate Matter	0.250	0.000	0.125	0
potassium ferricyanide	0.250	0.000	0.125	0
vanadium pentoxide	0.250	0.000	0.125	0
zinc protoporphyrin	0.250	0.000	0.125	0
Aflatoxin_B1	0.000	0.241	0.120	0
Arsenic	0.125	0.111	0.118	1
Mustard Gas	0.125	0.093	0.109	0
Selenium	0.125	0.093	0.109	0
Arbutin	0.125	0.056	0.090	0
Cadmium Chloride	0.125	0.056	0.090	0
Dibutyl Phthalate	0.125	0.056	0.090	0
Lead	0.125	0.056	0.090	0
Mercury	0.125	0.056	0.090	0
Rotenone	0.125	0.056	0.090	0
Sodium Azide	0.125	0.056	0.090	0
Uranium	0.125	0.056	0.090	0
Butylated Hydroxytoluene	0.125	0.037	0.081	0
Hypochlorous Acid	0.125	0.037	0.081	0
methyl cellosolve	0.125	0.037	0.081	0
Potassium Dichromate	0.125	0.037	0.081	0
quinoline	0.125	0.037	0.081	0
sodium arsenate	0.125	0.037	0.081	0
2-ethoxyethanol	0.125	0.019	0.072	0
3-xylene	0.125	0.019	0.072	0
4-tert-octylphenol	0.125	0.019	0.072	0
Acrolein	0.125	0.019	0.072	0
Atrazine	0.125	0.019	0.072	0
barium chloride	0.125	0.019	0.072	0
Beryllium	0.125	0.019	0.072	0
Butyric Acid	0.125	0.019	0.072	0
Calcium Chloride	0.125	0.019	0.072	0
Dioxins	0.125	0.019	0.072	1
eleostearic acid	0.125	0.019	0.072	0
kojic acid	0.125	0.019	0.072	0
Linuron	0.125	0.019	0.072	0
Manganese	0.125	0.019	0.072	0
mono-(2-ethylhexyl)phthalate	0.125	0.019	0.072	1
n-butoxyethanol	0.125	0.019	0.072	0
N,N,N',N'-tetrakis(2-pyridylmethyl)ethylenediamine	0.125	0.019	0.072	0
nickel sulfate	0.125	0.019	0.072	0
nonylphenol	0.125	0.019	0.072	0

octylphenol	0.125	0.019	0.072	0
osthol	0.125	0.019	0.072	0
Phosphorus	0.125	0.019	0.072	0
sodium selenate	0.125	0.019	0.072	0
Toluene 2,4-Diisocyanate	0.125	0.019	0.072	0
tributyltin	0.125	0.019	0.072	0
Trinitrotoluene	0.125	0.019	0.072	0
1,2,3,6,7,8-HxCDD	0.125	0.000	0.063	1
2,2',3',4,4',5-hexachlorobiphenyl	0.125	0.000	0.063	0
2,3',4,4',5-pentachlorobiphenyl	0.125	0.000	0.063	0
2,4,5-Trichlorophenoxyacetic Acid	0.125	0.000	0.063	0
3,4,5,3',4',5'-hexachlorobiphenyl	0.125	0.000	0.063	0
4-nonylphenol	0.125	0.000	0.063	0
acephate	0.125	0.000	0.063	0
Acids	0.125	0.000	0.063	0
aluminum fluoride	0.125	0.000	0.063	0
aluminum lactate	0.125	0.000	0.063	0
aluminum phosphide	0.125	0.000	0.063	0
Anabolic Agents	0.125	0.000	0.063	0
antimony oxide	0.125	0.000	0.063	0
arsenic acid	0.125	0.000	0.063	0
arsenic disulfide	0.125	0.000	0.063	0
arsenic trichloride	0.125	0.000	0.063	0
benz(a)anthracene	0.125	0.000	0.063	0
beryllium fluoride	0.125	0.000	0.063	0
beryllium sulfate	0.125	0.000	0.063	0
cadmium acetate	0.125	0.000	0.063	0
Chromium Alloys	0.125	0.000	0.063	0
chromium hexavalent ion	0.125	0.000	0.063	0
chromium oxide	0.125	0.000	0.063	0
Coal Tar	0.125	0.000	0.063	0
copper (N-2-hydroxyacetophenone)glycinate	0.125	0.000	0.063	0
cypermethrin	0.125	0.000	0.063	0
Diazinon	0.125	0.000	0.063	0
Dichlorodiphenyl Dichloroethylene	0.125	0.000	0.063	0
dicyclohexyl phthalate	0.125	0.000	0.063	0
diethyl maleate	0.125	0.000	0.063	0
dimethyl mercaptosuccinate	0.125	0.000	0.063	0
ethyl 6-(N-(2-chloro-4-fluorophenyl)sulfamoyl)cyclohex-1-ene-1-carboxylate	0.125	0.000	0.063	0
furan	0.125	0.000	0.063	0
gallium nitrate	0.125	0.000	0.063	0
Halogenated Diphenyl Ethers	0.125	0.000	0.063	0
Heptachlor Epoxide	0.125	0.000	0.063	1
Herbicides	0.125	0.000	0.063	0
mancozeb	0.125	0.000	0.063	0
methamidophos	0.125	0.000	0.063	0
methyl arachidonylfluorophosphonate	0.125	0.000	0.063	0
methyl isobutyl ketone	0.125	0.000	0.063	0
Mirex	0.125	0.000	0.063	1
nickel chloride	0.125	0.000	0.063	0
pentabromodiphenyl ether	0.125	0.000	0.063	1
PCB 180	0.125	0.000	0.063	1
Plant Oils	0.125	0.000	0.063	0
prochloraz	0.125	0.000	0.063	0
selenium oxide	0.125	0.000	0.063	0
sodium bichromate	0.125	0.000	0.063	0
sodium bisulfide	0.125	0.000	0.063	0
tin mesoporphyrin	0.125	0.000	0.063	0
titanium alloy (TiAl6V4)	0.125	0.000	0.063	0
titanium nitride	0.125	0.000	0.063	0
trimellitic anhydride	0.125	0.000	0.063	0
Vehicle Emissions	0.125	0.000	0.063	0

vinclozolin	0.125	0.000	0.063	0
vinylidene chloride	0.125	0.000	0.063	0
Formaldehyde	0.000	0.111	0.056	0
Asbestos,_Crocidolite	0.000	0.093	0.046	0
Cacodylic_Acid	0.000	0.074	0.037	0
Methylcholanthrene	0.000	0.074	0.037	0
Trichloroethylene	0.000	0.074	0.037	0
2,4-diaminotoluene	0.000	0.056	0.028	0
bis(tri-n-butyltin)oxide	0.000	0.056	0.028	0
Diethylhexyl_Phthalate	0.000	0.056	0.028	0
Potassium	0.000	0.056	0.028	0
2-nitrofluorene	0.000	0.037	0.019	0
4-(N-methyl-N-nitrosamino)-1-(3-pyridyl)-1-butanone	0.000	0.037	0.019	0
Arsenates	0.000	0.037	0.019	0
Clofibric_Acid	0.000	0.037	0.019	0
dimethylarsinous_acid	0.000	0.037	0.019	0
Dust	0.000	0.037	0.019	0
methylselenic_acid	0.000	0.037	0.019	0
uranyl_acetate	0.000	0.037	0.019	0
2-nitrotoluene	0.000	0.019	0.009	0
Acrylamide	0.000	0.019	0.009	0
alachlor	0.000	0.019	0.009	0
Antimony	0.000	0.019	0.009	0
Antimony_Potassium_Tartrate	0.000	0.019	0.009	0
Asbestos	0.000	0.019	0.009	0
Barium	0.000	0.019	0.009	0
bromochloroacetic_acid	0.000	0.019	0.009	0
butylbenzyl_phthalate	0.000	0.019	0.009	0
Chlordan	0.000	0.019	0.009	1
Chlorides	0.000	0.019	0.009	0
di-n-pentyl_phthalate	0.000	0.019	0.009	0
dicyclanil	0.000	0.019	0.009	0
diphenyltin_chloride	0.000	0.019	0.009	0
Diphosphates	0.000	0.019	0.009	0
Dithiothreitol	0.000	0.019	0.009	0
Endrin	0.000	0.019	0.009	0
Lithium_Chloride	0.000	0.019	0.009	0
Mercuric_Chloride	0.000	0.019	0.009	0
Metals,_Heavy	0.000	0.019	0.009	0
methylmercury_hydroxide	0.000	0.019	0.009	0
methylmercury_II	0.000	0.019	0.009	0
Methylnitronitrosoguanidine	0.000	0.019	0.009	0
Metribolone	0.000	0.019	0.009	0
monoisoamyl-2,3-dimercaptosuccinate	0.000	0.019	0.009	0
monomethylarsonous_acid	0.000	0.019	0.009	0
N-acetylsphingosine	0.000	0.019	0.009	0
N-nitrosomorpholine	0.000	0.019	0.009	0
perchlorate	0.000	0.019	0.009	0
phenylhydrazine	0.000	0.019	0.009	0
Piperonyl_Butoxide	0.000	0.019	0.009	0
Polychlorinated_Biphenyls	0.000	0.019	0.009	1
Radon	0.000	0.019	0.009	0
Soot	0.000	0.019	0.009	0
terephthalic_acid	0.000	0.019	0.009	0
tetraarsenic_tetrasulfide	0.000	0.019	0.009	0
tetrabutyltin	0.000	0.019	0.009	0
1,2,3,4,6,7,8-HpCDD	0.000	0.000	0.000	1
A1260	0.000	0.000	0.000	1
Agent orange	0.000	0.000	0.000	1
DDD	0.000	0.000	0.000	1
DDE	0.000	0.000	0.000	1
HCH	0.000	0.000	0.000	1
lidane	0.000	0.000	0.000	1

OCDD	0.000	0.000	0.000	1
organochlorines	0.000	0.000	0.000	1
organotins	0.000	0.000	0.000	1
oxychlordane	0.000	0.000	0.000	1
PBB153	0.000	0.000	0.000	1
PBBs	0.000	0.000	0.000	1
PBDE100	0.000	0.000	0.000	1
PBDE153	0.000	0.000	0.000	1
PBDE28	0.000	0.000	0.000	1
PBDE47	0.000	0.000	0.000	1
PBDE99	0.000	0.000	0.000	1
PBDEs	0.000	0.000	0.000	1
PCB153	0.000	0.000	0.000	1
PCDD/PCDF	0.000	0.000	0.000	1
PFHS	0.000	0.000	0.000	1
PFNA	0.000	0.000	0.000	1
trans-nonachlor	0.000	0.000	0.000	1

Table S5: List of genes associated with the four selected chemicals, arsenic, HCB, PFOA and TCDD after additional exploration of the possible pathogenesis carried out by extracting the curated chemical-gene-T2D interactions from the CTD database (n.d. : not determined for humans).

	**HUGO name**	**EntrezGene ID**
**HCB**	CAT	847
	FAS	355
	HMOX1	3162
	HP	3240
	IL6	3569
	IRS1	3667
	TIMP1	7076
	TNFRSF1A	7132
**PFOA**		
	AP3S2	10239
	ATF3	467
	ATP2A2	488
	C3	718
	CAT	847
	CPT1A	1374
	GCGR	2642
	GCK	2645
	GCKR	2646
	GPD2	2820
	HMOX1	3162
	HNF1B	6928
	HPX	3263
	ID1	3397
	IL6	3569
	LEPR	3953
	LIPC	3990
	NFKB1	4790

	PCX	n.d.
	PPARA	5465
	PPARG	5468
	PPARGC1A	10891
	PSMD6	9861
	SLC2A2	6514
	ST6GAL1	6480
	TNFRSF1A	7132
	UCP2	7351
**Arsenic**		
	ADIPOQ	9370
	ATF3	467
	CAT	847
	EDNRA	1909
	EDNRB	1910
	GCK	2645
	HMOX1	3162
	ID1	3397
	IL6	3569
	LEP	3952
	LEPR	3953
	NFKB1	4790
	PPARA	5465
	PPARGC1A	10891
	TNFRSF1A	7132
	UBE2E2	7325
**TCDD**		
	ADAMTS9	56999
	ADCY5	111
	ADIPOQ	9370
	AKT2	208
	AP3S2	10239
	ATF3	467
	ATP2A2	488
	ATP2A3	489
	C3	718
	CAT	847
	CPT1A	1374
	ECE1	1889
	EDN1	1906
	EDNRB	1910
	ENPP1	5167
	FAS	355
	GCK	2645
	GCKR	2646
	GPD2	2820
	GPX1	2876
	GRB14	2888

HK1	3098
HMOX1	3162
HNF1A	6927
HNF1B	6928
HNF4A	3172
HP	3240
HPX	3263
ID1	3397
IL6	3569
INS1	n.d.
IRS1	3667
IRS2	8660
KCNJ11	3767
LEP	3952
LEPR	3953
LIPC	3990
MAEA	10296
NFKB1	4790
NOTCH2	4853
PAX4	5078
PAX6	5080
PCX	n.d.
PEPD	5184
PPARA	5465
PPARG	5468
PPARGC1A	10891
PROX1	5629
PTPN1	5770
RETN	56729
S100A6	6277
SLC2A1	6513
SLC2A2	6514
SLC2A4	6517
ST6GAL1	6480
TCF7L2	6934
THADA	63892
TIMP1	7076
TNFRSF1A	7132
TNFRSF1B	7133
UBE2E2	7325
UCP2	7351
VEGFA	7422
WFS1	7466
ZFAND3	60685

Table S6: Number of proteins connected to diseases and pathways for the four selected chemicals. For example, of the 5515 proteins included in the GeneCards database, 206 are known to be associated with diabetes mellitus. PFOA is

connected to 27 proteins, but only 23 are present in GeneCards, of which 12 are linked to diabetes mellitus.

Chemical (connected/ present in database)	GeneCards (5515)		Reactome (5283)	KEGG (6176)
	Diabetes mellitus (206)	Niddm (228)	Diabetes pathway (309)	Type II diabetes (48)
PFOA (23/27)	12	13	1	2
TCDD (56/65)	26	26	2	8
Arsenic (16/16)	9	7	1	2
HCB (8/8)	5	3	0	1
